# Effects of extensive bleeding in pigs on laboratory biomarkers

**DOI:** 10.48101/ujms.v126.6914

**Published:** 2021-05-18

**Authors:** Anders Larsson, Gunnar Strandberg, Miklós Lipcsey, Mats Eriksson

**Affiliations:** a Department of Medical Sciences, Uppsala University, Uppsala, Sweden; b Department of Surgical Sciences, Uppsala University, Uppsala, Sweden; c Department of Cardiothoracic Surgery and Anesthesiology, Uppsala University, Uppsala, Sweden; d Hedenstierna Laboratory, Department of Surgical Sciences, Uppsala University, Uppsala, Sweden

**Keywords:** Analyte, Biochemistry, Circulation, Experimental, Exsanguination, Shock

## Abstract

**Background:**

During hemorrhage and resuscitation, clinical and laboratory monitoring is useful to guide further management. However, acute changes in the biochemistry due to blood loss and subsequent crystalloid fluid resuscitation have not been fully studied.

**Materials and methods:**

Twelve anesthetized, juvenile pigs were used. Atraumatic exsanguination, corresponding to a total blood loss of 40%, was performed through a catheter and completed 2 h after initiation of the experiment. Arterial samples were analyzed by point-of-care testing and venous samples were analyzed. Oxygen delivery was calculated.

**Results:**

Shortly after 40% hemorrhage and concomitant fluid supplementation, there were significant reductions in arterial hemoglobin and hematocrit (approximately 25%, respectively). Oxygen delivery was less than half of the baseline value. Lactate in arterial blood was more than doubled after 40% exsanguination. On average, no other clinically significant changes in any of the analytes were observed, but interindividual dispersion was pronounced.

**Conclusions:**

Acute exsanguination was associated with decreased hemoglobin and hematocrit levels and increased lactate levels but limited effects on the other biomarkers that were studied. Increased levels of biomarkers in severely bleeding patients could indicate tissue damage and the source should be further investigated.

## Introduction

When extensive bleeding occurs, it is important to be able to distinguish between the effects of the hemorrhage and fluid resuscitation *per se*, and organ damage, either as a consequence of trauma or as a result of hypoperfusion. In cases of severe hemorrhage, when blood products are not readily available, resuscitation with crystalloid fluids is currently the first-line treatment. When severe bleeding occurs outside the hospital, optimal fluid therapy is not always an option. In the extra-hospital setting, merely basic interventions may be at hand, with the intention of maintaining cardiac output and oxygen delivery as well as restoring perfusion pressure, which requires adequate cardiac preload. On admission to an emergency department, blood sampling for guidance of continued care is a natural step regardless of whether the circulatory stability has been achieved or not. Further resuscitation should be guided by a goal-directed strategy (1), including a transfusion protocol, since extensive hemorrhage will result in loss of coagulation factors (2). Identification and management of bleeding in patients pre-treated with anticoagulant agents is a challenging mission since adequate therapy may be delayed if such treatment is overlooked or not properly managed.

The extent to which acute bleeding affects the results of laboratory analyses frequently used in emergencies is not sufficiently studied. Thus, it might be questioned whether an abnormal laboratory test result is caused by organ damage or a consequence of exsanguination and its management.

In a previous study (3), we reported that intraosseous samples, analyzed by point-of-care technology, during experimental hemorrhage rapidly reflect changes in arterial and venous blood samples although precision in the intraosseous samples was limited.

Little is known regarding the effect of more extensive blood loss on organ-specific biomarkers – which analyses are possibly affected and to what extent? For instance, does a traumatic liver injury with subsequent bleeding manifest itself as elevated levels of alanine aminotransferase (ALT) (4) or could such an elevation be masked by hemorrhage and fluid resuscitation? It would for obvious reasons not be ethically justifiable to design a clinical study in humans where a hypovolemic shock condition is induced.

In order to further evaluate the effect of severe and rapid hemorrhage and fluid resuscitation on the results of laboratory analyses, this experimental study was conducted. Since this study focuses on acute extensive hemorrhage, we utilized, when possible, point-of-care technology, in order to mimic an emergency requiring urgent intervention and decision-making.

## Materials and methods

Twelve juvenile male Yorkshire/Norwegian Landrace pigs, mean weight 24.6 ± 3.2 kg, were included in this study, which was carried out in a university hospital animal research laboratory. The animals in this study represent a subset of a previous approval, obtained from the local animal ethics committee (application no. C155/14). All animals were handled according to the guidelines of the Swedish Board of Agriculture and the European Convention of Animal Care.

The animals were anesthetized shortly upon arrival from the breeder with an intramuscular injection of 6 mg/kg of tilétamin-zolazepam and 2.2 mg/kg of xylazin. Peripheral venous access was established, and anesthesia was deepened by injections of 100 mg of ketamine and 20 mg of morphine, after which a tracheotomy was performed and mechanical ventilation with a Servo U^®^ ventilator (Maquet Critical Care, Solna, Sweden) was commenced. Anesthesia was maintained with a continuous infusion of pentobarbital at 8 mg/kg/h and morphine at 0.48 mg/kg/h, both dissolved in a 2.5% glucose solution containing 70 mmol Na^+^/L. Muscle relaxants were not used.

Arterial, pulmonary artery, and central venous catheters were inserted via a right-sided neck incision. A 13.5 Fr central dialysis catheter was inserted via a left-sided neck incision. A urinary catheter was inserted via a minor laparotomy. Invasive pressures, respiratory parameters, pulse oximetry, Electrocardiogram (ECG), diuresis, and temperature were registered continuously during the experiment. Mean arterial pressure (MAP) and mean pulmonary arterial pressure (MPAP) were continuously monitored through the arterial and the Swan–Ganz catheters, respectively. Cardiac output was assessed by the thermodilution method, where 10 mL of ice-cold saline was injected intravenously, using the thermistor in the Swan–Ganz catheter. The average value from at least three serial measurements was registered. Cardiac index (CI [L × min^-1^ × m^-2^]) was calculated as cardiac output/body surface area (weight^0.67^ × 0.112) (5).

After the initial preparation and a short period of stabilization, central venous and arterial samples were taken and analyzed with an i-STAT^®^ handheld point-of-care analyzer equipped with EG7+ and CG4+ cartridges (Abbott Point of Care, Princeton, NJ, USA). pH, arterial partial pressure of carbon dioxide (PaCO_2_), arterial partial pressure of oxygen (PaO_2_), sodium, potassium, ionized calcium (Ca^++^), hematocrit, and lactate were analyzed. Secondary calculated parameters, provided by the analyzer, were hemoglobin oxygen saturation (SaO_2_), bicarbonate (HCO_3_^-^), base excess (BE), and hemoglobin (Hb). Analyses were performed immediately after sampling, and the entire process took approximately 10 min. Central venous samples were transferred to test tubes and centrifuged, and plasma was analyzed for aspartate aminotransferase (AST), ALT, alkaline phosphatase (ALP), cholesterol, triglycerides, high-density lipoprotein (HDL)-cholesterol, uric acid, pancreas amylase, lactate dehydrogenase (LDH), magnesium, total plasma protein, total bilirubin, γ-glutamyltransferase (γ-GT), glucose, creatine kinase (CK), and creatinine on a Mindray BS380 (Mindray, Shenzhen, China) using reagents from Abbott Laboratories. DO_2_ was calculated as CO × ([1.31 × Hb × SaO_2_ × 0.01] + [PaO_2_ × 0.025]) (6). We decided to calculate DO_2_ since reduced tissue oxygenation is crucial in the development of organ damage and therefore seen as an arbitrary indication of hypovolemic shock.

After these baseline measurements, 20% of the calculated blood volume (BV) of 67 mL/kg (5) was removed from the circulation through the dialysis catheter, followed by a stabilization period, after which, an additional 20% of initial BV was removed, that is, 40% in total, which corresponds to 27 mL/kg (5). The two steps of exsanguination and corresponding sampling were completed 2 h after the start of the experimental procedure. Total fluid administration during the entire experiment was 15 mL/kg/h (anesthetic drugs were administered at 8 mL/kg/h and a supplementary balanced electrolyte solution [Ringer-Acetate™] was administered at 7 mL/kg/h).

Average levels and measures of dispersion for all analytes at baseline and 40% hemorrhage were calculated. Means, medians, and standard deviations were calculated using the Microsoft^®^ Excel^®^ version 16.16.25. 2018. Interquartile range (IQR) and Wilcoxon’s one-tailed rank sum test were calculated using Social Science Statistics. *P* < 0.05 was considered significant.

## Results

As a consequence of extensive hemorrhaging, *noticeable* circulatory derangements were observed (Table 1). MAP and CI were significantly reduced after both 20 and 40% exsanguination (*P* < 0.01; all four calculations).

**Table 1:** Heart frequency (HF; bpm), mean arterial pressure (MAP; mm Hg), mean pulmonary arterial pressure (MPAP; mm Hg), and cardiac index (CI; L/min/m^2^) at baseline, and after 20% exsanguination and 40% exsanguination, respectively.

	Baseline		20% hemorrhage		40% hemorrhage	
	Median	IQR	Median	IQR	Median	IQR
HF (bpm)	101	146–77	97	119–87	109	127–99
MAP (mm Hg)	83	91–69	53	57–44	40	52–34
MPAP (mm Hg)	18	18–16	14	16–13	15	17–13
CI (L/min/m^2^)	2.9	4.2–2.3	1.8	2.1–1.7	1.5	1.9–1.2

Concentrations of pH, PaCO_2,_ PaO_2,_ standard bicarbonate (HCO_3_^-^), base excess, SaO_2_, sodium, potassium, and ionized calcium in arterial blood samples were not significantly lower after 40% exsanguination. This was in contrast to both hemoglobin and hematocrit, which were significantly reduced (*P* < 0.05) after 40% hemorrhage compared to the baseline concentrations. Relative reductions in hemoglobin were approximately 25 at 40% hemorrhage. Corresponding changes in hematocrit levels were in the same order.

Lactate increased from 1.7 to 4.2 mmol/L (*P* < 0.05) after 40% hemorrhage, whereas no other significant changes were found. Ionized calcium decreased from 1.34 ± 0.04 to 1.30 ± 0.05 mmol/L after 40% blood loss (n.s.). Glucose level increased from 7.5 ± 0.8 to 8.6 ± 1.5 mmol/L after 40% blood loss (n.s.). Arterial analytes are presented in Table 2 and venous analytes are presented in Table 3.

**Table 2:** Initial values and absolute and relative changes, respectively, after 40% exsanguination and their maximal/minimal alterations in arterial blood samples.

Analyte	Initial value	Max/min	Absolute change	Max/min	Relative change %	Max/min
pH	7.53	7.59/7.46	−0.01	0/−0.01	−0.13	0.13/−0.35
SaO_2_ (%)	100	100/99	0	0/−1	0	0/−1
PaO_2_ (kPa)	23.1	38/15	0.25	2/−1.1	1	10/−5
PaCO_2_ (kPa)	4.8	6.0/4.0	−0.3	−0.04/−0.9	−6	−1/−15
HCO_3_^−^ (mmol/L)	31	34/28	−3	−1/−5.5	−9	−3/−20
Base excess (mmol/L)	7.5	11/4	−2.5	−1/−6	−31	−9/−150
Lactate (mmol/L)	1.7	5.3/0.8	2.23	3.8/1.2	131	151/33
Sodium (mmol/L)	138	140/134	−2.5	−1/−4	−2	−1/−3
Potassium (mmol/L)	3.8	4.4/3.5	0.6	1/0.3	17	23/9
iCa (mmol/L)	1.3	1.4/1.3	−0.045	−0.03/−0.09	−3	−3/−26
Hemoglobin (g/L)	79	88/65	−18.5	−21/−14	−23	−22/−26
Hematocrit (%)	23	26/19	−6	−4/−7	−25	−21/−29

**Table 3:** Initial values and absolute and relative changes, respectively, after 40% exsanguination and their maximal/minimal alterations in venous blood samples.

Analyte	Initial value	Max/min	Absolute change	Max/min	Relative change %	Max/min
Cholesterol (mmol/L)	1.8	2.3/1.5	−0.3	0.1/−0.5	−19	4/−24
HDL-cholesterol (mmol/L)	1.8	2.3/1.4	−0.4	0/−0.6	−22	−1/−28
Triglycerides (mmol/L)	0.2	0.9/0.1	0.2	0.4/−0.4	93	368/−2
AST (mmol/L)	0.4	0.4/0.3	−0.1	0/−0.1	−11	7/−21
ALT (mmol/L)	1.1	1.5/1.1	−0.2	0/−0.4	−22	24/2
ALP (mmol/L)	1.9	2.2/0.9	−0.3	0.2/−0.4	−15	16/20
γ−GT (mmol/L)	0.2	0.3/0.1	0.0	0.4/−0.1	−18	112/41
Glucose (mmol/L)	7.6	8.8/5.7	1.1	3.6/0.7	15	41/10
Urate (mmol/L)	9.1	16.9/3.2	4.0	8.0/−0.1	38	61/10
Pancreatic amylase (mmol/L)	27.5	34.7/14.4	−4.8	2.2/−8.8	−20	16/−26
Creatine kinase (mmol/L)	9.0	36.3/2.4	−1.1	19.7/−31.2	−13	322/−86
Creatinine (µmol/L)	81.5	98.0/65.0	8.5	26/−12	11	28/−13
LDH (umol/L)	1.0	1.4/0.6	−0.1	0.2/−0.5	−9	27/−36
Magnesium (mmol/L)	0.8	0.9/0.5	0.0	0.2/−0.2	−3	21/−31
Plasma protein (g/L)	39.2	43.6/27.6	−8.1	10.8/−13.0	−21	28/−34
Bilirubin; total (µmol/L)	0.6	0.8/0.3	−0.1	0.2/−0.2	−12	35/−26

Exsanguination did not significantly lower the measured amount of cholesterol, triglycerides, HDL-cholesterol, uric acid, pancreatic amylase, CK, creatinine, LDH, magnesium, total protein content, total bilirubin, AST, ALT, ALP, γ-GT, and glucose in central venous samples.

Oxygen delivery (Table 4) was markedly reduced after 40% exsanguination. The post-hemorrhage decrease was statistically significant compared to the initial value (*P* < 0.05).

**Table 4:** Oxygen delivery (DO_2_) was significantly (*P* < 0.05) reduced after 40% hemorrhage as compared to baseline.

Analyte	Initial value	Max/min	Absolute change	Max/min	Relative change %	Max/min
DO_2_ (mL/min)	310	483/170	−175	−47/−323	−62	−23/−81

## Discussion

This study focuses on the accuracy of laboratory test results after acute extensive bleeding and moderate fluid supplementation. Recent studies on this topic are sparse, and biochemistry analyzers are undergoing development which provides further rationale to examine the extent to which hemodilution may have an impact on the validity of bioanalytical responses. Not only trauma-associated factors, such as blood loss, dilution, and consumption of coagulation factors, but also time-dependent factors, for example, development of hypothermia and acidosis, may contribute to the development of traumatic coagulopathy (2, 7). Severe bleeding is often coexisting with damage to specific tissues. In those situations, it is crucial to be able to distinguish whether an abnormal test result is due to the bleeding *per se* or caused by organ injury.

Neither hemoglobin nor hematocrit was lowered by as much as 40%, respectively, compared to baseline values, which may be explained by sequential dilution of blood during exsanguination. We were only able to detect the significant effects of hemorrhage on DO_2_, hematocrit, and hemoglobin. In theory, hemorrhage and resuscitation causing a 25% reduction in hemoglobin and hematocrit might lead to similar decreases in the concentrations of other analytes. Arterial lactate increased significantly which is not surprising since lactic acidemia is known to occur in hemorrhagic shock (8) and more generally in critical conditions characterized by decreased DO_2_ (9). Ionized calcium (coagulation factor IV [FIV]) was not significantly lowered (approximately 4%). This is of interest, since analysis of ionized calcium is of major importance in extensive hemorrhage as FIV needs to be supplemented during heavy bleeding (10).

Exsanguination also includes the loss of plasma and plasma proteins. A 25% reduction of plasma should lead to lower levels of several of the studied plasma proteins if they were not replenished from the extracellular space. The median concentrations of liver enzymes were reduced by approximately 20% after a 40% exsanguination indicating that part of the loss was substituted from extravascular sources, although elevations due to organ damage cannot be excluded. In a study by Marques et al. (11), designed to evaluate precision of continuous noninvasive assessment of blood hemoglobin in hemorrhaging humans, 12 volunteers were bled (10 mL/kg for 15 min) while Ringer’s lactate solution was infused at 30 mL/kg for 20 min. Blood hemoglobin was assessed by pulse oximetry and compared to arterial hemoglobin content. The non-invasive continuous hemoglobin monitoring was assessed by Bland–Altman analysis for repeated measurements; calculated bias between the two methods was 1.08 ± 0.82 g/dL, and 95% levels of agreement were between −0.5 and 2.6.

Our aim to elucidate the possible effect of extensive hemorrhage on laboratory variables gave rise to more extensive testing than motivated in an emergency, where a life-threatening blood loss is at hand, since we wanted to test the concept as far as possible.

This study has several limitations. The most obvious one is the fact that this study was carried out in a limited number of healthy animals in an experimental setting. Male juvenile pigs were used in an attempt to standardize the material. It cannot be excluded that this might have had some minor impact on our results, since circulatory parameters were better preserved in sexually immature female piglets, when compared to their male counterparts in an experimental model of hemorrhage and cardiac arrest (12). Major differences between the referenced study (12) and our study are that our pigs were not subjected to cardiac arrest and cardio-pulmonary resuscitation. Furthermore, if a gender-specific impact would be present, we would most likely have overestimated the consequences of hemorrhage and fluid supplementation.

Another limitation is that the bleeding was performed in two sequences even if the bleedings may be regarded as one acute event with concomitant fluid supplementation. Also, the circulating BVs within the pigs were not measured by a BV test, but estimated from previous data (5). It must not be overlooked that some variables were lowered due to blood loss and that absence of significant reductions may be due to statistical type II errors.

## Conclusion

In conclusion, 40% exsanguination was associated with lowered hemoglobin and hematocrit levels as well as increased lactate levels in arterial blood samples, markedly lowered global oxygen delivery (DO_2_), and circulatory derangement. Elevated levels of biomarkers may indicate organ damage, rather than insufficient tissue oxygenation. However, the overall clinical relevance of hemorrhagic shock and fluid supplementation on the plasma levels of biochemical markers seems insufficiently investigated.

## References

[1] Rossaint R, Bouillon B, Cerny V, Coats TJ, Duranteau J, Fernández-Mondéjar E, et al. The European guideline on management of major bleeding and coagulopathy following trauma: fourth edition. Crit Care. 2016;20:100. doi: 10.1186/s13054-016-1265-x 27072503 PMC4828865

[2] Spahn DR, Bouillon B, Cerny V, Duranteau J, Filipescu D, Hunt BJ, et al. The European guideline on management of major bleeding and coagulopathy following trauma: fifth edition. Crit Care. 2019;23:98. doi: 10.1186/s13054-019-2347-3 30917843 PMC6436241

[3] Strandberg G, Larsson A, Lipcsey M, Eriksson M. Comparison of intraosseous, arterial, and venous blood sampling for laboratory analysis in hemorrhagic shock. Clin Lab. 2019;65. doi: 10.7754/Clin.Lab.2019.181214 31307157

[4] Kaptanoglu L, Kurt N, Sikar HE. Current approach to liver traumas. Int J Surg. 2017;39:255–9. doi: 10.1016/j.ijsu.2017.02.015 28193544

[5] Hannon JP, Bossone CA, Wade CE. Normal physiological values for conscious pigs used in biomedical research. Lab Anim Sci. 1990;40:293–8. 2162986

[6] Dunn J-OC, Mythen MG, Grocott MP. Physiology of oxygen transport. BJA Educ. 2016;16:341–8. doi: 10.1093/bjaed/mkw012

[7] Harrois A, Hamada SR, Duranteau J. Fluid resuscitation and vasopressors in severe trauma patients. Curr Opin Crit Care. 2014;20:632–7. doi: 10.1097/MCC.0000000000000159 25340381

[8] Pearce FJ, Connett RJ, Drucker WR. Extracellular-intracellular lactate gradients in skeletal muscle during hemorrhagic shock in the rat. Surgery. 1985;98:625–31. 4049240

[9] Rashkin MC, Bosken C, Baughman RP. Oxygen delivery in critically ill patients. Relationship to blood lactate and survival. Chest. 1985;87:580–4. doi: 10.1378/chest.87.5.580 3987371

[10] Moore HB, Tessmer MT, Moore EE, Sperry JL, Cohen MJ, Chapman MP, et al. Forgot calcium? Admission ionized-calcium in two civilian randomized controlled trials of prehospital plasma for traumatic hemorrhagic shock. J Trauma Acute Care Surg. 2020;88:588–96. doi: 10.1097/TA.0000000000002614 32317575 PMC7802822

[11] Marques NR, Kramer GC, Voigt RB, Salter MG, Kinsky MP. Trending, accuracy, and precision of noninvasive hemoglobin monitoring during human hemorrhage and fixed crystalloid bolus. Shock. 2015;44:45–9. doi: 10.1097/SHK.0000000000000310 25521537

[12] Semenas E, Nozari A, Wiklund L. Sex differences in cardiac injury after severe haemorrhage and ventricular fibrillation in pigs. Resuscitation. 2010;81:1718–22. doi: 10.1016/j.resuscitation.2010.08.010 20817375

